# First person – Ashley Gendreau

**DOI:** 10.1242/bio.062132

**Published:** 2025-07-10

## Abstract

First Person is a series of interviews with the first authors of a selection of papers published in Biology Open, helping researchers promote themselves alongside their papers. Ashley Gendreau is first author on ‘ How octopuses use and recruit additional arms to find and manipulate visually hidden items’, published in BIO. Ashley is a research assistant in the lab of Dr Roger Hanlon at Marine Biological Laboratory, Woods Hole, MA, USA, investigating marine biology and animal behavior, with a focus on how animals sense, move through and interact with their environments.


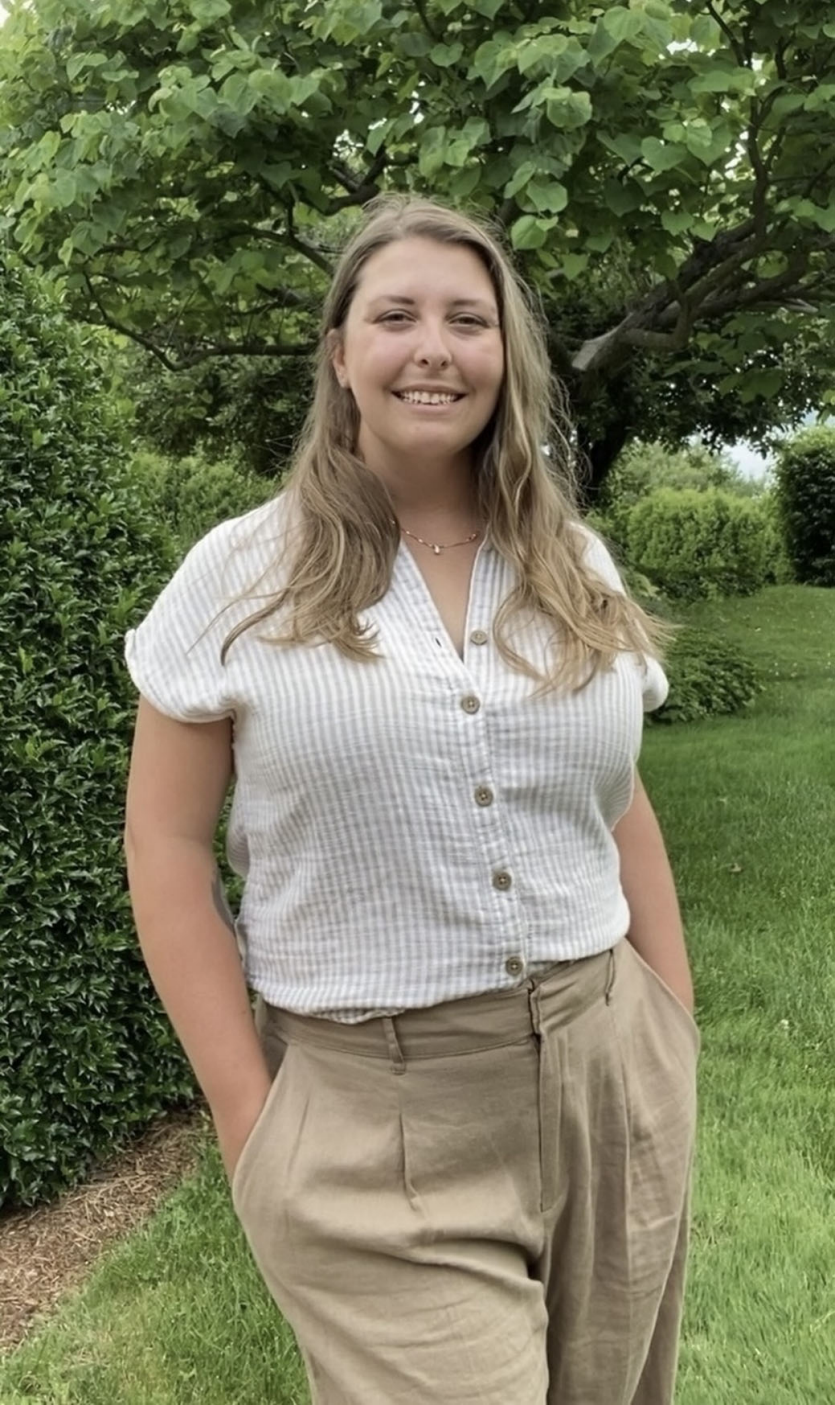


**Ashley Gendreau**

**Describe your scientific journey and your current research focus**

I've always been captivated by the mysteries of the unknown and the thrill of discovery – two pillars that, to me, define the very spirit of science. It just took a winding path for me to realize where those passions could lead. After serving in the military and spending several years in non-scientific industries, the COVID-19 shutdown left me at a crossroads. I used that moment to ask a simple question: what did my childhood-self dream of doing? The answer was clear – marine biology. I returned to school, earning a BSc in Marine Biology from the University of Rhode Island, and soon after had the opportunity to join Dr Roger Hanlon's lab at the Marine Biological Laboratory. Today, as a research assistant, I study the whole-animal behavior of my favorite invertebrate – the octopus! My current work explores how these remarkable animals coordinate their eight arms and hundreds of individual suckers when tackling complex tasks in the lab. By linking detailed behavioral observations to the octopus's still-mysterious neuroanatomy, we hope to uncover new principles of distributed control that could inspire advances in fields from neuroscience to soft robotics.

By linking detailed behavioral observations to the octopus's still-mysterious neuroanatomy, we hope to uncover new principles of distributed control that could inspire advances in fields from neuroscience to soft robotics

**Who or what inspired you to become a scientist?**

My inspiration for becoming a scientist began after an unforgettable ‘Dissect-a-Squid Night’ in elementary school. That night our cafeteria was transformed into a miniature lab: each student received a squid, a basic anatomy guide, and the glow of 20,000 Leagues Under the Sea projected on the wall. As I carefully teased apart that seemingly alien animal – mapping out how its parts worked together and relating it to my own anatomy – I felt the first spark of scientific curiosity and a lifelong love of cephalopods. I even went home with its beak as a trophy, convinced I'd just unlocked a secret of the deep.

**How would you explain the main finding of your paper?**

Our work explored how octopuses use their eight semi-autonomous arms to locate and manipulate objects in an environment where vision is limited. We found that octopuses are incredibly flexible in their arm behavior. Their eight arms not only act independently with remarkable similarity but also coordinate together, frequently but not exclusively in neighboring pairs, to manipulate an object more efficiently. Typically, three to five arms work simultaneously, a ‘divide-and-cooperate’ strategy that lets the animal explore swiftly while keeping other limbs free for new tasks. Our findings suggest that despite having a highly decentralized nervous system, octopuses exhibit specialized and repeatable patterns of arm use, offering new insights into sensorimotor control in animals with nontraditional anatomies.

**What are the potential implications of this finding for your field of research?**

Octopuses have captivated public and scientific attention alike, but much about their nervous system remains a mystery. Our findings help bridge the gap between behavior and neuroanatomy by revealing how motor control is distributed – and yet organized – in an animal with a decentralized brain dictating every move even when vision is obscured. Understanding these principles could not only advance our knowledge of cephalopod biology but also inform the development of soft-bodied robotics and decentralized control systems. By studying how octopuses coordinate their arms in complex, visually occluded tasks, we're uncovering fundamental strategies that could inspire neuroscience, engineering, and beyond.

**Figure:**
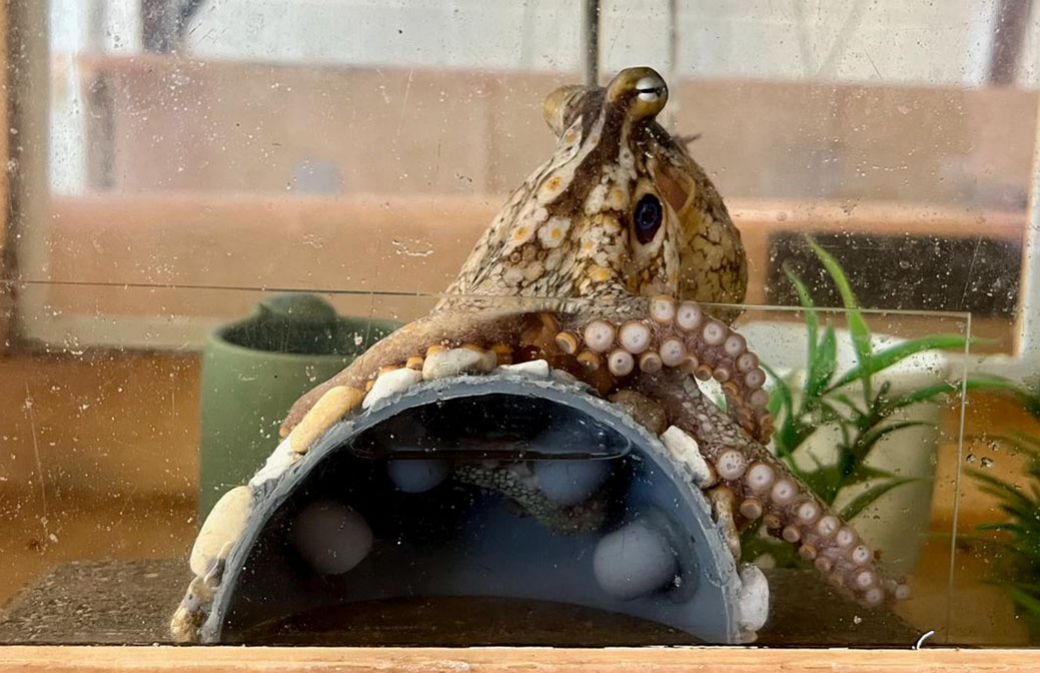
A California two-spot octopus (*Octopus bimaculoides*) using multiple arms to coordinate exploration and manipulation of an item within a visually occluded, 3D-printed experimental dome.

**Which part of this research project was the most rewarding?**

The most rewarding part has been sharing our findings with others. Whether it's fellow scientists, students, or curious members of the public, people are genuinely excited to hear about octopus behavior – and often surprised by how much we're still discovering. I love seeing that spark of curiosity ignite in others and being part of a conversation that extends far beyond the lab.

**What do you enjoy most about being an early-career researcher?**

What I enjoy most is the constant learning. Every day brings something new – whether it's picking up a new skill from a mentor, troubleshooting a method on my own, or reframing a question in light of unexpected results. I also feel incredibly lucky to be based at the Marine Biological Laboratory, where collaboration and innovation are woven into the culture. Being surrounded by and learning from passionate scientists at all stages of their careers is both inspiring and energizing as I continue to shape my own path in research.

…every experience – whether it's a perfect fit or not – teaches you something valuable

**What piece of advice would you give to the next generation of researchers?**

Say yes to as many opportunities as you can, especially early on and across different areas of research. Finding your niche can feel overwhelming at first, but every experience – whether it's a perfect fit or not – teaches you something valuable. If you stay curious and open every door, you'll eventually find the space where your skills, interests, and passions align.

**What's next for you?**

Next, I plan to begin a new round of research exploring how octopuses use their arms when faced with more complex tasks – pushing the boundaries of what we know about their abilities. Looking further ahead, I hope to pursue graduate studies that connect animal behavior research with broader environmental sciences. I'm also passionate about science communication and envision a future where I can play a role in ocean conservation education – whatever form that may take.
